# Molecular, Population, and Clinical Aspects of Lipoprotein(a): A Bridge Too Far?

**DOI:** 10.3390/jcm8122073

**Published:** 2019-11-27

**Authors:** Natalie C. Ward, Karam M. Kostner, David R. Sullivan, Paul Nestel, Gerald F. Watts

**Affiliations:** 1 School of Public Health, Curtin University, Perth 6102, Australia; natalie.ward@uwa.edu.au; 2 School of Medicine, University of Western Australia, Perth 6009, Australia; 3 Department of Cardiology, Mater Hospital, Brisbane 4104, Australia; k.kostner@uq.edu.au; 4 School of Medicine University of Queensland, Brisbane 4072, Australia; 5 Medical School, The University of Sydney, Sydney 2006, Australia; david.sullivan@sydney.edu.au; 6 Charles Perkins Centre, The University of Sydney, Sydney 2006, Australia; 7 Department of Biochemistry, Royal Prince Alfred Hospital, Sydney 2050, Australia; 8 Baker Heart & Diabetes Institute, Melbourne 3004, Australia; paul.nestel@baker.edu.au; 9 Department of Cardiology, The Alfred Hospital, Melbourne 3004, Australia; 10 Lipid Disorders Clinic, Department of Cardiology, Royal Perth Hospital, Perth 6000, Australia

**Keywords:** lipoprotein(a), atherosclerotic cardiovascular disease, calcific aortic valve disease

## Abstract

There is now significant evidence to support an independent causal role for lipoprotein(a) (Lp(a)) as a risk factor for atherosclerotic cardiovascular disease. Plasma Lp(a) concentrations are predominantly determined by genetic factors. However, research into Lp(a) has been hampered by incomplete understanding of its metabolism and proatherogeneic properties and by a lack of suitable animal models. Furthermore, a lack of standardized assays to measure Lp(a) and no universal consensus on optimal plasma levels remain significant obstacles. In addition, there are currently no approved specific therapies that target and lower elevated plasma Lp(a), although there are recent but limited clinical outcome data suggesting benefits of such reduction. Despite this, international guidelines now recognize elevated Lp(a) as a risk enhancing factor for risk reclassification. This review summarises the current literature on Lp(a), including its discovery and recognition as an atherosclerotic cardiovascular disease risk factor, attempts to standardise analytical measurement, interpopulation studies, and emerging therapies for lowering elevated Lp(a) levels.

## 1. Introduction

There is now significant evidence to support an independent causal role for elevated lipoprotein(a) (Lp(a)) as a risk factor for atherosclerotic cardiovascular disease (ASCVD). Plasma Lp(a) concentrations are predominantly determined by genetic factors. Research into Lp(a) has been hampered by an incomplete understanding of its metabolism and proatherogeneic properties, including a lack of available animal models. Furthermore, a lack of standardized assays to measure Lp(a) and no universal consensus on optimal plasma levels in different patient populations remain significant obstacles. In addition, there are currently no approved specific and potent therapies to target and reduce Lp(a) although recent limited clinical outcome data suggest benefits of such reduction. Despite this, most international guidelines now recognize elevated Lp(a) as a risk-enhancing factor that warrants further investigation. This review summarises the current Lp(a) literature including its discovery and recognition as a risk factor, attempts to standardise analytical methods, consideration of ethnic differences, as well as new treatments to lower elevated Lp(a) levels. 

The literature search included the search terms “lipoprotein(a)” and “Lp(a)” in combination with diagnosis, treatment, pathogenesis, and guidelines, with additional key references provided by authors of this article.

## 2. History and Renaissance

Lp(a) was discovered more than 50 years ago by geneticist Kare Berg, when it was initially believed to be a blood group antigen (a) [1]. Subsequent cloning of the cDNA encoding the apolipoprotein(a) (apo(a)) portion revealed its homology with the fibrinolytic proenzyme, plasminogen [2], although distinct structural differences were later recognized, including the disappearance of the tail domain and the first three kringle domains [3]. Over the last decade, several studies have established the important, independent, and causal role of elevated Lp(a) in both atherosclerotic cardiovascular disease (ASCVD) and calcific aortic valve disease (CAVD) [4].

## 3. Molecular Aspects

### 3.1. Physical Chemistry

**Figure 1:**
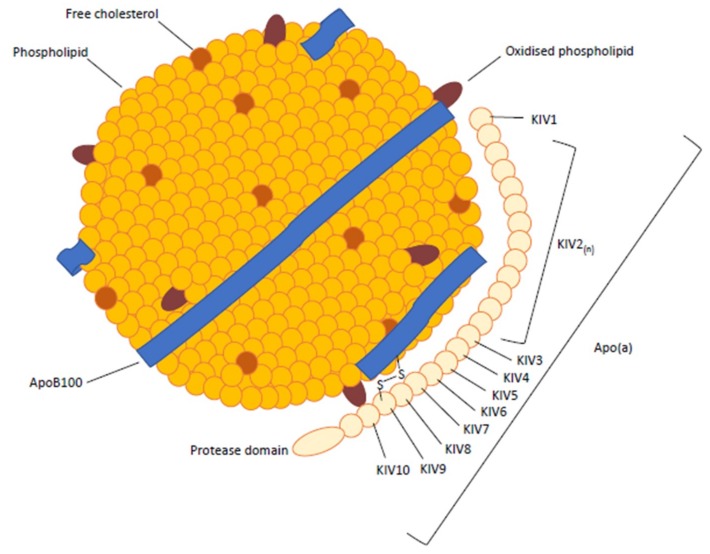
Structure lipoprotein(a). ApoB = apolipoprotein B, apo(a) = apolipoprotein a and KIV = kringle repeat.

Lp(a) is a highly polymorphic particle that is structurally similar to but larger than Low-density lipoprotein (LDL) [5]. The major difference is the presence of a hydrophilic glycoprotein, termed apo(a), which is noncovalently and covalently bound (via a disulfide bridge) to apolipoprotein B-100 (apoB-100) [5,6,7,8]. Within the apo(a), there are multiple repeated copies of sequences homologous to plasminogen’s kringle 4 domain (KIV), followed by a kringle 5-like (KV) domain and a protease-like domain (Figure 1). The kringle IV-like units differ in their amino acid sequence, with KIV2 present at varying copy numbers (ranging from 12 to 51), while all others are present as single copies. The KIV domains also contain lysine binding sites of varying strength [3,5]. Within the KIV7 and KIV8 domains, weak lysine binding sites are required for noncovalent interactions with the lysine domains of apoB-100, while the covalent disulphide linkage occurs with a unique, unpaired cysteine residue in KIV9 [9]. The KIV10 domain contains a strong lysine binding site that mediates interactions between apo(a) and biological substrates, including fibrin and oxidised phospholipid [9]. In addition, the molecular weight of the apo(a) (400 to 700 kDa) determines density and mobility characteristics [7], which results in >30 isoforms of Lp(a) [8], with 80% of individuals carrying two different-sized isoforms [10]. 

### 3.2. Genetics 

**Figure 2:**
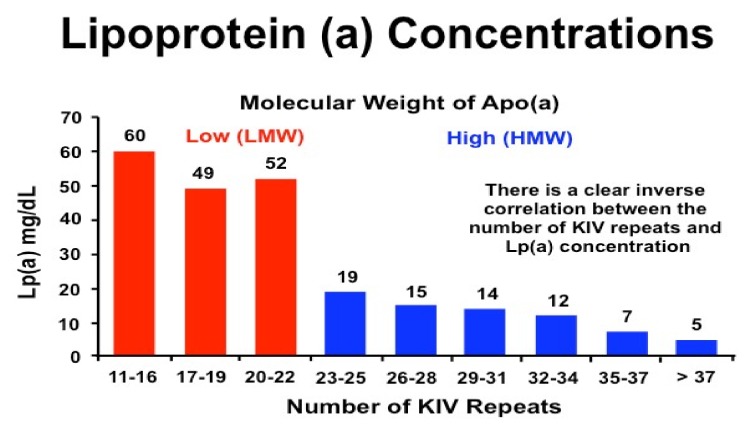
Association between lipoprotein(a) concencentration and isoform size (Reproduced from Reference [21]). Apo(a) = apolipoprotein (a), Lp(a) = lipoprotein (a), LMW = low molecular weight, HMW = high molecular weight, KIV = kringle number repeats.

Lp(a) is highly heritable, with plasma levels predominantly (90%) determined by variation in the Lipoprotein (a) (*LPA*) gene on chromosome 6q26-27 [11,12,13]. This gene encodes apo(a) and is highly expressed in the liver [6,14,15]. The gene evolved from the plasminogen gene (*PLG*) and is only present in primates (old world monkeys and apes), European hedgehogs, and humans; regulation of its expression remains unclear [14,16]. The KIV domain within the *LPA* gene has diversified into ten types, with KIV2 existing in multiple copies (2 to >40 repeats, with a repeat size of 5.6 kB), and genetic variants in this locus can have a pronounced influence on Lp(a) concentrations. Lp(a) concentrations are variable within the different apo(a) isoform sizes, and this is dependent on the KIV2 copy number repeats. Individuals with a low number of repeats have a small apo(a) isoform and higher plasma Lp(a) concentrations in contrast to those with high (>22) repeats and, therefore, only large apo(a) isoforms [11,17] (Figure 2). Lp(a) concentrations are also dependent on genetic variation including pentanucleotide repeats in the promoter region, variants that affect RNA splicing, and single nucleotide polymorphisms (SNPs) within the structural and functional domains [8,14,15,16]. Overall, copy number variation, which determines apo(a) isoform size, accounts for 25–50% of variability and is inversely correlated with plasma levels, while low frequency SNPs, including single and repeat polymorphisms in varous regions of the *LPA* gene, account for ~35% of variability [15,16,18]. Genetic variants of Apolipoprotein e gene (*APOE*) have also been shown to affect plasma Lp(a) levels [19], with the *APOE ε2* variant strongly associated with low Lp(a) concentrations, although this did not appear to modify any association with myocardial infarction or aortic valve stenosis [20]. 

### 3.3. Biology

Lp(a) biosynthesis has four main processes: transcription of *LPA*, protein translation, movement through the secretory pathway, and assembly of the Lp(a) particles. Transcription is regulated by an IL-6 responsive element, a DR-1 promotor element, an Ets motife, and multiple cyclic adenosine monophosphate (cAMP) responsive elements located in the *LPA* promoter region. Secretion is largely regulated by apo(a) size, with larger isoforms retained for longer in the endoplasmic reticulum, leading to increased proteasomal degradation. Assembly of Lp(a) remains controversial, with some studies suggesting intracellular and others supporting extracellular assembly of apo(a) and LDL [3,9].

As yet, no physiological function for Lp(a) has been conclusively established [22], although early research suggested a role in bleeding and wound healing and it is now known to be the major carrier of oxidised phospholipids [3]. Plasma levels are mainly determined by *LPA* allele size, with an inverse correlation between apo(a) isoform size and plasma Lp(a) concentration [3] (Figure 2). Plasma levels are generally resistant to diet or various physiological and environmental factors, including age, sex, fasting state, or physical activity, but vary between different ethnic groups [6,14,22]. Obstructive liver disease and high plasma bile salt concentration are associated with extremely low Lp(a) levels, which led to the discovery of an farnesoid X receptor (FXR) signalling mechanism in the regulation of apo(a) and, therefore, Lp(a) [8]. Pregnancy, menopause, and use of hormone replacement therapy also appear to influence plasma Lp(a) levels, although there is considerable heterogeneity between studies [22]. Other hormones, including testosterone and thyroxine may reduce Lp(a) levels, while acute phase events, including myocardial infarction, may transiently increase or decrease Lp(a) levels, although this remains controversial [23]. Renal function also appears to influence Lp(a) levels, although this may be related to clearance, with elevated Lp(a) levels in patients with renal impairment, which are inversely correlated with glomerular filtration rate [24]. 

As the apo(a) component has no traditional lipid-binding domains and its hydrophilic nature means it can exist in the aqueous phase, Lp(a) is able to interact with the vascular endothelium and cell receptors to facilitate divergent effects on vascular phenotypes [25]. Thus, in addition to passive diffusion through the endothelial surface, Lp(a) may also accumulate in vascular tissue and be retained in subendothelial surfaces [25]. This can lead to induction of cell adhesion molecules, reduced barrier function in vascular endothelial cells with endothelial dysfunction, smooth muscle cell proliferation and migration, as well as induction of inflammatory cytokine expression and apoptosis [9]. The clearance of Lp(a) from plasma remains unclear, with the LDL receptor thought to play only a modest role, although the frequently elevated Lp(a) concentration in patients with familial hypercholesterolaemia (FH) suggest some LDL receptor involvement. Other possibilities include catabolism by the kidney via scavenger, toll-like, lectins, LDL receptor-related protein (LRP), or plasminogen receptors or through proteolytic cleavage of apo(a) [3,6,22,26,27]. Biochemical studies suggests that these receptors associate with Lp(a) via the apoB, apo(a), or oxidised phospholipid component, although their specific involvement in Lp(a) clearance from the circulation remains unclear [27].

## 4. Epidemiological and Observational Studies

### 4.1. Cohorts

Despite early suggestions that Lp(a) was not an independent risk factor in the absence of elevated LDL-c levels [28,29], many epidemiological studies confirm a positive association between circulating Lp(a) levels and risk of ASCVD [29,30,31,32,33,34,35,36]. An early study in patients with FH found higher levels of Lp(a) in those with coronary heart disease (CHD) compared with those without, which was independent of traditional risk factors [37]. Meta-analysis of long-term prospective studies, including statin trials, reveal a continuous, independent, and modest association of Lp(a) concentration and risk of CHD and stroke [30,38,39]. Furthermore, Lp(a) and LDL-c have been demonstrated to be independently associated with cardiovascular disease (CVD) risk [40], with only a modest attenuation in risk when LDL-c levels are lowered and Lp(a) remains elevated [41]. Interestingly, the risk associated with premature acute coronary syndrome appears to be higher in younger and middle-aged populations compared with those aged >60 years [42]. Supporting this, a recent prospective population-based study has found a positive association between Lp(a) levels and 10-year CVD incidence, particularly in those <45 years of age. Adherence to a Mediterranean diet and healthy ageing, as determined by the successful ageing index, were found to decrease and to potentially mediate the adverse effect of Lp(a) on CVD risk. Again, this observation was more significant in younger adults [43]. Large population studies also support elevated Lp(a) as a risk factor for first atherothrombotic events [44]. A recent retrospective observational study found that elevated Lp(a) (≥25 mg/dL) was associated with a higher prevalence of thin-cap fibroatheroma, suggestive of plaque vulnerability, which was further enhanced in patients who also had elevated LDL-c [45]. In people with preexisting CVD, individual studies are inconsistent. Post hoc analysis from proprotein convertase subtilisin/kexin type 9 (PCSK9) intervention studies provide some evidence that lowering of Lp(a) in addition to LDL-c is beneficial. This is supported by Mendelian randomisation studies; however, the estimates and how much of a reduction is required vary [46]. A recent analysis of two studies from the Danish population have found that lowering Lp(a) by 50 mg/dL reduces short CVD by 20% in a secondary prevention setting [47]. However, meta-analysis of secondary prevention trials reveal that elevated Lp(a) predicted major adverse cardiovascular events (MACE), albeit with considerable heterogeneity among studies. Such heterogeneity can be attributed to differences in study design, statistical power, and index event bias, although it is also important to recognise issues relating to sample storage, measurement methodology, possible changes in Lp(a) levels as a result of clinical events, and difference in clinical management between primary and secondary prevention [44]. Although there is limited data on the association between Lp(a) levels and cardiovascular mortality, a recent study has investigated individuals from two prospective studies of the Danish general population. This analysis, which included information on Lp(a) concentration, KIV2 repeat number, and rs10455872 genotype, on elevated Lp(a) was associated with an increased risk of both cardiovascular and all-cause mortality. This association with high Lp(a) concentration was due to low KIV2 repeats rather than a higher cholesterol content, suggesting that the mortality effect of elevated Lp(a) is not explained solely by its cholesterol content [48].

Within the general population, elevated Lp(a) levels have also been associated with an increased risk of aortic valve stenosis. Within the group with levels >90 mg/dL, there was a predicted threefold increased risk [49]. This was further confirmed in a prospective Mendelian randomisation study and case–control cohort study, suggestive of a causal association [50] and in asymptomatic patients with FH [51]. In a prospective study of patients with mild-to-moderate aortic stenosis, those with the highest levels of Lp(a) and oxidised phospholipids had more rapid progression of stenosis and the highest risk of valve replacement and death [52]. More recently, the level of Lp(a) and its oxidised phospholipid content has been associated with an accelerated progression of calcific aortic valve stenosis [53]. In patients with aortic stenosis, Lp(a) and oxidised phospholipid increased valve calcification activity, increased progression of valvular computed tomography calcium score, and was associated with an increased risk for aortic valve replacement and death [54]. 

Lp(a) may also be an independent risk factor for peripheral artery disease (PAD) [55], with the InCHIANTI community-based study demonstrating a cross-sectional association between Lp(a) and lower-limb PAD [56]. The EPIC-Norfolk prospective population study found that Lp(a) levels were associated with future PAD and CAD events, independent of LDL-c levels [57].

### 4.2. Cross National Study

Epidemiological studies have demonstrated that Lp(a) levels can be modestly related to ethnicity [58,59]. In BiomarCaRE, regional differences in Lp(a) levels were seen within the European population [60]. The INTERHEART study of different ethnicities revealed significant variation in Lp(a) concentration and size between ethnic groups. Africans were found to have the highest concentration of Lp(a) and the smallest isoform size, while Chinese patients had the lowest Lp(a) concentrations and largest isoform sizes. Higher Lp(a) concentrations were associated with an increased risk of myocardial infarction in all ethnicities but particularly in South Asians and Latin Americans, independent of traditional risk factors. Isoform size did not significantly contribute to risk, particularly after adjustment for concentration, although there was an inverse association between isoform size and circulating levels [61]. In a study of Chinese patients, elevated Lp(a) was associated with an increased risk of acute myocardial infarction, even when LDL-c levels were normal [62]. Interestingly, the Multi-Ethnic Study of Atherosclerosis found that Lp(a) was associated with risk of heart failure in white, but not black, Hispanic, or Chinese participants. Significant race interactions were observed, and Lp(a) was further related to a greater risk of heart failure with preserved ejection fraction in white participants. This was independent of aortic valve disease, although the exact mechanism for these associations remains unclear [63]. 

### 4.3. Statin-Treated Patients

The JUPITER trial revealed that, in white patients treated with potent statin therapy, Lp(a) was a significant determinant of residual risk, although the magnitude of risk reduction in those treated with rosuvastatin was similar amongst patients with high or low Lp(a) [64]. In a meta-analysis of statin-treated patients, elevated baseline and on-treatment Lp(a) levels were independently associated with increased CVD risk [40]. A recent meta-analysis suggests statin treatment may increase Lp(a) levels by 10–20%, with atorvastatin associated with a dose-dependant increase in Lp(a) [6,65]. Interestingly, a recent study in patients initiating or already on stable statin treatment found that statins affect Lp(a) levels differently, dependent on the apo(a) phenotype. Specifically, statins appeared to increase Lp(a) levels exclusively in patients with the low molecular weight apo(a) phenotype [66].

### 4.4. Type 2 Diabetes Patients

In contrast to ASCVD and CAVD, there appears to be an inverse association between Lp(a) concentration and risk of type 2 diabetes. The Bruneck population-based prospective study suggested a 12% higher risk of type 2 diabetes for a one standard deviation lower concentration of log Lp(a). Subsequent meta-analysis of four prospective cohort studies found that the risk of type 2 diabetes was higher in those with the lowest two quintiles of Lp(a) concentration, with the highest risk in those with a Lp(a) < 7 mg/dL [67]. Risk of cardiovascular events in patients with diabetes, however, appears to be positively associated with plasma Lp(a) levels, with elevated Lp(a) independently associated with the presence and severity of CAD in type 2 diabetic patients [68]. In BiomarCaRE, Lp(a) was associated with an increased risk for major coronary events and CVD in patients with diabetes [60]. A recent multi-centre study has found that elevated Lp(a) levels were associated with a significantly higher risk of cardiovascular events in stable CAD patients with prediabetes or diabetes compared to those with normal glucose metabolism [69]. The biracial Atherosclerosis Risk in Communities study found that elevated Lp(a) levels in Caucasian individuals with diabetes or prediabetes was associated with additional increased risk of ASCVD [70]. The reason for the inverse association between plasma Lp(a) levels and risk of diabetes remains unclear and may be related to isoform size or possibly to the inverse association with triglycerides [71]; however, this warrants further investigation. 

## 5. Genetic Studies

### 5.1. Genome-Wide Association

Analysis of individual variants in 63,743 cases and 130,681 controls revealed that the most potent genetic association with CAD was the *LPA* locus, more so than variants related to LDL, PCSK9, or 9p21 [72]. The first genome-wide association study to confirm the importance of Lp(a) as a risk factor indentified the rs10455872 and rs3798220 variants as having odds ratios of 1.70 and 1.92, respectively. Both variants were associated with elevated Lp(a), reduced copy number of *LPA*, and small Lp(a) size. Furthermore, when combined, these variants had an odds ratio of 2.57 [73]. Genome-wide association studies have also identified rs10455872 SNP to be significantly associated with aortic-valve calcification and incident aortic stenosis across multiple ethnic groups [74]. This association was specific for aortic valve disease, with no genome-wide associations observed for mitral valve calcification [25]. These findings were replicated in three Danish cohorts, where elevated Lp(a) levels were associated with an increased risk of myocardial infarction. Number of KIV2 repeats was also correlated with plasma levels of Lp(a) and myocardial infarction risk, with elevated Lp(a) as determined by genetic variance associated with a hazard ratio of 1.22 per doubling of Lp(a) [75]. In a separate analysis of two of these Danish cohorts, high Lp(a) levels, specifically as a result of a low KIV2 number of repeats and thus small apo(a) isoform size, was associated with high risk of both cardiovascular and all-cause mortality [48]. Conversely, a highly frequent variant in the KIV2 region that is strongly associated with reduced plasma Lp(a) concentrations was associated with reduced cardiovascular risk in a European population [76]. A recent analysis of single-base polymorphisms in the KIV2 region has identified 14 different loss-of-function and splice-site mutations as well as >100 missense variants. Subsequent reanalysis of individuals from 26 different populations using this genetic map revealed differences in the frequencies of coding variants between different populations, which may account for the large ethnic and individual differences in plasma Lp(a) levels [17]. This supports an earlier finding of sequence variation within the KIV2 region between European, Asian, and African populations [77]. 

### 5.2. Mendelian Randomisation

The causal role of Lp(a) in myocardial infarction, ASCVD, and CAVD is strongly supported by Mendelian randomization studies. This approach, which is generally not affected by confounding and reverse causation, is particularly valuable with Lp(a) due to the existance of single genetic variants, which explain ~28% of the variation in plasma Lp(a) levels. The most important of these is likely the KIV2 copy number variant [18]. Supporting this are studies that have demonstrated that genetically determined smaller apo(a) size and increased Lp(a) plasma concentration are independent and causal risk factors for a range of cardiovascular conditions, including coronary heart disease (CHD) [78], heart failure [79], aortic valve calcification [74], and stenosis [49]. Recent mendelian randomization analysis has also examined the required Lp(a) lowering effect size that is required to reduce CHD outcomes. A Lp(a) reduction of 65.7 mg/dL was estimated to have the same effect as a 38.67 mg/dL lowering of LDL-c, although this was determined from observed estimated effects of SNP on Lp(a) concentration and is thus influenced by the standardization of the assay used [80].

## 6. Analysis of Lp(a) Levels

### 6.1. Reference Values

Distribution of plasma Lp(a) concentration is dependent on the population studied, with circulating concentrations varying up to 1000 fold within the same patient population (ranging from <0.1 mg/dL to >300 mg/dL or from 2.5 nmol/L to 750 nmol/L). In Caucasians, plasma levels are similar for men and women and skewed, with ~20% of the population having levels >50 mg/dL. In the lowest quintile, Lp(a) concentrations are <5 mg/dL. Levels are lowest in non-Hispanic Caucasians, Chinese, and Japanese; slightly higher in Hispanics; and highest in Blacks [81]. While plasma concentrations of Lp(a) vary considerably, the risk of CHD is thought to be proportionally associated with the absolute increase in Lp(a) mass concentration. Its been postulated that Lp(a) must be lowered by ~100 mg/dL to achieve the same CHD risk reduction as lowering LDL-c by 38.67 mg/dL [82]. However, it must be noted that, while the risk between LDL-c and CVD is linear, the risk associated with Lp(a) is continuous and curvilinear and is driven by extreme values [83]. Evidence is accumulating to support elevated plasma concentrations of >125 nmol/L or >50 mg/dL as an independent, causal risk factor for a number of CVDs [9]. Nevertheless, substantial risk reduction is achieved in those with levels >80 mg/dL at baseline or those in the highest decile (~100 mg/dL).

### 6.2. Bioanalytical Issues

Measurement of circulating Lp(a) levels remains a challenge due to the complex structure of the molecule, in particular the highly polymorphic nature of the apo(a), with reproducible and reliable results difficult to obtain due to the variation in isoform size [9,84]. A large number of assays report Lp(a) values as mass concentrations (mg/dL) compared with particle concentrations (nmol/L), with a lack of standardisation across laboratories [9,84]. This places significant limitations on the measurement in that variable amounts of each of the components of Lp(a) may be different among patients, skewing the results. As a result, reporting Lp(a) values in molar concentrations of apo(a) as nmol/L, which quantitates total apop(a) particle number and is not dependent on the molecular weight of Lp(a), is now recommended [85]. The majority of Lp(a) assays also use antibody-based approaches, which rely on multi-epitope binding of polyclonal antibodies raised against the target antigen. Because Lp(a) has a large variable copy number in the KIV2 region, this affects the capture efficiency of the larger isoforms compared with smaller isoforms [9,84]. The International Federation of Clinical Chemistry and Laboratory Medicine (IFCC) has recommended the use of a standardised reference material to calibrate Lp(a) assays and to improve the reproducibility between methods [86]. Both the IFCC and the World Health Orgnisation (WHO) approved a primary reference material. However, when this was used to calibrate different commercial assays that were testing a range of Lp(a) concentrations and apo(a) isoform sizes, considerable heterogeneity remained [87,88]. The bias created by the variation in repeat units in differently sized apo(a) isoforms typically manifests as an underestimation of the levels with small Lp(a) isoforms and an overestimation with the large Lp(a) isoforms [88]. A recent comparison of six commercially available Lp(a) immunoassays has further highlighted how the biases between assays differ significantly across the clinically relevant concentration range in a nonlinear manner, highlighting the need for harmonisation of the Lp(a) assays [89]. The gold standard for measurement of isoform size is high-resolution sodium dodecyl sulfate-agarose gel electrophoresis (SDS-AGE). This method is capable of determining each Lp(a) isoform present in a plasma sample, although the method is time consuming, laborious, and not routinely performed in the clinic [90]. Additional methods, involving ultra-performance liquid chromatography/mass spectrometry (UPLC/MS), which can determine both concentration and isoform size, are being developed, although these are limited by an inability to determine the number of KIV2 domains independently due to each *LPA* allele [91]. More recently, a newer immunoturbidometric system that still uses the primary reference material but has a five-point calibration system has revealed minimal apo(a) isoform size bias. This method also expresses Lp(a) concentration in nanomoles per litre [92,93]. 

## 7. Pathobiology: Atherosclerotic Cardiovascular Disease and Calcific Aortic Valve Disease

### 7.1. Risk Factor Status

The National Heart, Lung, and Blood Institute working group acknowledges that pathophysiological, epidemiological, and genetic studies provide strong evidence that Lp(a) is a causal mediator of both ASCVD and CAVD [94]. Although these two conditions have a distinct aetiology, their risk factor profiles along with the underlying cellular and molecular pathologies have common overlap [3]. Lp(a) is thought to contribute to the increased risk through three main mechanisms: the atherogenic nature of its LDL-like moiety, the thrombogenic and anti-fibrinolytic effects of its apo(a) moiety, and the pro-inflammatory effects of its oxidised phospholipid content predominantly derived from LDL [6]. What is critical is recognition of the strong genetic control of Lp(a) which results in elevated exposure from birth and thus cumulative burden of risk throughout a lifespan [95]. 

### 7.2. Atherosclerotic Cardiovascular Disease (ASCVD)

In addition to the pro-atherogenic role of its components, Lp(a) is thought to contribute to the development of ASCVD through its effects on vascular cells. This includes the induction of adhesion molecules, which has been shown to be apo(a) dependent as well as stimulated through the lysine dependent binding of Lp(a) [9,96]. Additional effects include an increased permeability of endothelial cells exposed to Lp(a) via disruption of the cytoskeleton and adheren junctions [97] and enhanced endothelial and smooth muscle cell proliferation and migration, a process that is critical in the development of atherosclerosis [9]. Finally, the oxidised phospholipids associated with Lp(a) have been shown to accelerate the inflammatory processes that lead to lesion formation, with further stimulation of apoptoic and pro-inflammatory cascades in monocytes, macrophages, and endothelial and smooth muscle cells, once incorporated into the vessel wall [9]. 

### 7.3. Calcific Aortic Valve Disease (CAVD)

The mechanisms for the role of Lp(a) in CAVD are still unclear, although it is due in part to the oxidised phospholipid content, specifically the high content of lysophosphatidylcholine, which can be transformed by autotaxin to lysophosphatidic acid. This then promotes the deposition of hydroxyapatite of calcium within the aortic valve, resulting in inflammation and mineralisation [14,98]. Recent in vitro work has demonstrated that Lp(a) induces oesteogenic differentiation of valvular interstitial cells, which was mediated by oxidised phospholipid [54]. Additional mechanisms include inflammation of the aortic valve leaflets, where Lp(a) binds to the denuded endothelial surface of the leaflets via its lysine binding sites and delivers proinflammatory molecules. The induced inflammation may further contribute via fibrosis leading to accelerated aortic valve thickening and loss of pliability, stenosis, and systolic orifice area reduction [15,25]. 

### 7.4. Thrombosis and Fibrinolysis

Given the high homology between apo(a) and plasminogen, it was originally suggested that Lp(a) may act as a modulator of blood clotting and fibrinolysis [14]. The in vitro effects of Lp(a) on platelet aggregation are inconsistent and vary depending on the agonist used and its concentration [99]. Within the circulation, Lp(a) has been shown to associate with platelet activating factor-acetylhydrolase (PAF-AH), an enzyme which inhibits PAF-induced platelet activation in a nonspecific manner [100]. Lp(a)-induced inhibition of fibrinolysis may increase risk of thrombosis, which at sites of plaque rupture may lead to increased risk of myocardial infarction and ischaemic stroke. Thrombosis at sites of turbulent blood flow may also contribute to both atherosclerotic and valvular aortic stenosis [88]. The true nature of the role of Lp(a) in thrombosis remains unclear, predominantly because, although elevated Lp(a) predisposes to thrombotic events in the aterial tree, these events are generally confounded by underlying atherosclerosis [101]. Furthermore, the evidence supporting a prothrombotic role for Lp(a) has largely been seen in vitro, with no conclusive observations seen in humans [101]. 

## 8. Clinical Aspects

### 8.1. Clinical Practice Guidelines

Recent guidelines from the American Heart Association (AHA) and American College of Cardiology (ACC) (Table 1) recognise elevated Lp(a) as a “risk-enhancing factor” in the development of ASCVD, supporting earlier guidelines from the European Atherosclerosis and Cardiology Societies [102,103]. Other groups, including the Canadian Cardiovascular Society and the Mighty Medic Group suggest Lp(a) might aid risk assessment in patients at high risk or with premature CVD/CAD, with Lp(a) levels <30 mg/dL considered normal [104,105]. The AHA/ACC guidelines suggest that Lp(a) levels ≥125 nmol/L (≥50 mg/dL) are considered high risk [103]. However, a recent scientific statement from the National Lipid Association (NLA) suggests that the 80th percentile in predominantly Caucasian US populations is ~100 nmol/L and ~150 nmol/L in African Americans, although it is unclear whether a different risk threshold should be applied, as levels are generally two- to fourhold higher in African Americans compared to Caucasians [84,88]. Newly released 2019 guidelines from the European Atherosclerosis and Cardiology Societies, however, now underplay the importance of elevated Lp(a). Whilst the guidelines recommend that Lp(a) measurement be considered at least once in each adult person’s lifetime to assist with risk stratification, particularly in those considered at moderate risk, they focus only on people with extremely elevated levels (>180 mg/dL or >430 nmol/L) who they suggest may have a lifetime risk of ASCVD equivalent to that of heterozygous FH [106]. The newly released HEART-UK consensus statement on Lp(a) also supports the measurement of Lp(a) levels in patients with a personal or family history of premature ASCVD, those with FH or genetic dyslipidaemia (familial combined hypercholesterolaemia) or CAVD, as well patients with a first degree relatives who have raised Lp(a) (>200 nmol/L). The statement suggests the cardiovascular risk conferred by Lp(a) is determined by its serum concentration, with 32–90 nmol/L equivalent to minor risk, 90–200 nmol/L equivalent to moderate risk, 200–400 nmol/L equivalent to high risk, and with >400 nmol/L equivalent to very high risk [107] (Table 1). 

### 8.2. Who to Test?

While screening for elevated Lp(a) levels is not routinely undertaken, measurement may be considered valuable in premature onset ASCVD, where elevated levels of Lp(a) are both more common and associated with more advanced atherosclerosis [108,109]. While the cost-effectiveness of screening and testing for Lp(a) remains to be shown, indications for its measurement are a family history of premature ASCVD or a personal history of ASCVD not explained by major risk factors [23,103]. Several national and international guidelines recommend Lp(a) testing if a patient has documented ASCVD (particularly with recurrent events on optimal lipid-lowering therapy), severe hypercholesterolaemia, FH, or premature ASCVD in the absence of traditional risk factors [88] (Table 1). Paradoxically, Lp(a) levels are lower in patients with type 2 diabetes [110]. In paediatric FH, elevated Lp(a) appears to be more predictive than elevated LDL-c for early onset disease in family members [111]. In cascade screening for FH, testing for elevated Lp(a) is effective for identifying relatives with high Lp(a) and, therefore, elevated risk of ASCVD. In patients with either FH or elevated Lp(a), there was a significantly increased risk of an ASCVD event or death, with the greatest risk seen in FH patients with elevated Lp(a), independent of conventional risk factors [112]. Elevated Lp(a) is also independently associated with future CHD death in patients with chronic kidney disease [113,114], and early studies suggest accelerated coronary atherosclerosis in heart transplant patients with elevated Lp(a) [115]. Of great importance is the frequency with which elevated Lp(a) concentrations are found among young male patients suffering their first myocardial infarction and in whom the only apparent risk factor is elevated Lp(a). In contrast, in a recent report of three cohorts of women who suffered cardiac events, Lp(a) appeared to be predictive only when LDL-c concentrations were also elevated [116].

### 8.3. How to Treat?

Currently, no therapeutic agents that specifically target and reduce elevated Lp(a) levels are available. Treatments are hindered by Lp(a) having no enzyme activity or receptor functions to target, and its high circulating levels mean antibody-based approaches would be unsafe and costly due to the large mass of immune complexes that would need to be cleared and to high amounts of antibody required [25]. Statin treatment does not appear to have any effect, and recent meta-analysis suggests they may increase circulating levels by 10–20% [6,65]. Other pharmacological therapies including niacin, microsomal triglyceride transfer protein (MTP) inhibitors, and cholesteryl ester transfer protein (CETP) inhibitors all lower Lp(a) levels to varying degrees; however, none has been specifically tested in randomised controlled trials and their clinical use remains limited [22,117]. Niacin, which has been shown to reduce Lp(a) by up to 30% [118], reduced CVD events in the Coronary Drug Project in 1975 [119], but its clinical use remains limited. Early studies indicated a role for hormones, including estrogen and testosterone, but these are not considered standard treatments [120,121]. A meta-analysis revealed significant reductions in Lp(a) following treatment with tamoxifen, a selective estrogen receptor modulator that is used to treat breast cancer [122]. Several natural products have also been proposed as promising Lp(a) lowering therapies, but their widespread use requires further investigation [123]. The strongest evidence that lowering Lp(a) reduces CVD risk comes from aphersis trials. A longitudinal multicentre cohort study with combined lipid apheresis and lipid-lowering therapy in patients with extremely high levels of Lp(a) demonstrated a reduction in MACE of >80% [124]. A prospective observational multicentre study from Germany also demonstrated a significantly reduced incidence of CVD events in patients with elevated Lp(a) treated with apheresis [125] which was confirmed in a 5-year prospective follow-up of the cohort [126]. Apheresis, however, is limited by high cost and patient burden, and its use is restricted to specific countries and patient populations. Furthermore, its use in specifically reducing risk associated with Lp(a) elevation is unknown due to concomitant reductions in LDL-c [127].

Large clinical outcomes studies in patients treated with PCSK9 monoclonal antibodies (PCSK9 mAbs) revealed reductions in plasma levels of Lp(a) by up to 30% [128]. In the FOURIER trial, evolocumab significantly reduced Lp(a) levels with the greatest reduction seen in those with the highest baseline levels. There was also a significant positive association between baseline Lp(a) levels and risk of cardiovascular events, irrespective of LDL-c levels, with the highest clinical benefit observed in patients who had the highest baseline levels of Lp(a). Significant reductions in CVD events (>30%) with evolocumab were observed among those with baseline Lp(a) levels >80 mg/dL, in whom the Lp(a) concentration halved [117].

In contrast, ODYSSEY OUTCOMES revealed that it was the extent of reduction in Lp(a) and not baseline value or magnitude of LDL-c reduction that predicted the degree of benefit seen with alirocumab (Bittner et al. presented at the 18th International Symposium on Atherosclerosis, Toronto Canada, 12, June, 2018). These promising results, however, are underscored somewhat by the ANITSCHKOW study, which found that patients with very high Lp(a) levels treated with evolocumab had only a 14% reduction in Lp(a) levels, which resulted in persistent Lp(a) elevation and no significant impact on arterial inflammation [129]. Furthermore, a pooled analysis of ten ODYSSEY phase 3 studies comparing alirocumab with placebo found that the PCSK mAb resulted in a median change in Lp(a) levels of 25.6%. This was associated with a 12% relative risk reduction in MACE, although this was no longer significant when adjusted for changes in LDL-c [130]. A recent study investigating the addition of PCSK9 mAbs to background niacin therapy found a ~15% further reduction in Lp(a) compared to niacin treatment alone [131].

### 8.4. What is New in Therapy?

The most successful therapies developed to date are the RNA-targeting treatments, including antisense oligonucleotides (ASOs) and small interfering RNA (siRNA). IONIS-APO(a)_Rx_ is an ASO that binds to the exon 24–25 splice site of the mature apo(a) transcript that results in dose-dependent decreases (39.6%, 59.0%, and 77.8%) in plasma Lp(a) levels over 4 weeks, with similar reductions in the amount of associated oxidised phospholipids [132,133]. A newly developed ASO, IONIS-APO(a)-L_Rx_, was found to be considerably more potent than its predecessor, with significant dose-dependent reductions in Lp(a) observed at lower doses [133]. A third phase II dosing and safety study of IONIS-APO(a)-L_Rx_ in patients with hyperlipoproteinemia(a) and CVD has recently been completed with results pending (NCT03070782). Novartis, which is developing the agent now known as TQJ230, is planning a phase III cardiovascular outcome trial in patients with existing CVD (NCT04023552), with recruitment set to begin in January 2020.

AMG890 is a newly developed therapy that specifically targets *LPA* RNA. A Phase I study investigating a single ascending dose to evaluate safety, tolerability, pharmacokinetics, and pharmacodynamics in people with elevated Lp(a) is currently underway, with an expected completion date of August 2020 (NCT03626662). Another alternative approach to reduce Lp(a) includes targeting the farnesoid X receptor [134]. Studies in transgenic mice have demonstrated a significant reduction in plasma concentration and hepatic expression of human *APOA* in response to FXR activation. This may be related to bile acid production, which activate the FXR receptor [135].

## 9. Future Challenges: Resarch Questions

The National Heart, Lung, and Blood Institute convened a working group to identify the challenges research faces in understanding the role of Lp(a) in ASCVD and CAVD. Many of these challenges include the lack of research funding, inadequate animal models, the lack of standardised methods for analysis, and an incomplete understanding of the mechanisms underlying both the role of Lp(a) in disease and current treatments to lower its levels [94]. Increased awareness of Lp(a) in the clinical setting and a greater understanding of the increased and/or residual risk that elevated Lp(a) contributes to CVD is important for ongoing management of patients [136]. In addition, there is also a need to better understand the biology of Lp(a), including its site of assembly, its route, and tissue source of catabolism and clearance, as well as a need for randomised controlled trials of treatments that specificially target Lp(a) in secondary prevention. This is particularly important given the significant residual risk that remains in patients with elevated Lp(a) despite reduced LDL-c [44]. In addition, Lp(a) and its role in the development of ASCVD needs to be incorporated into major guidelines, particularly as new treatments are developed and new trials are reported [92]. Studies and guidelines also need to address the role of Lp(a) and its treatments in various high-risk ethnic groups, including non-Caucasian populations and people with diabetes. Lastly, the role of Lp(a) in the development of CAVD [137] strongly supports the need for trials of apo(a)-targeting ASOs and the developing siRNA therapies in these populations.

## 10. Summary and Conclusions: A Bridge Too Far for Prime-Time Clinical Practice?

Genetic epidemiology proffers the best current evidence for the causal role of Lp(a) in ASCVD [29,30,31,32,33,34,35,36]. However, no clinical trial has demonstrated that selective reduction in elevated Lp(a) reduces the incidence of ASCVD events, diminishing the level of evidence and strength of recommendation for the routine measurement of Lp(a), as reflected by some international guidelines. Should one be nihilistic about Lp(a) or can we make some recommendations aligned with those of the NLA and HEART-UK? On the basis of the evidence reviewed, including expert opinion, we consider the quality of evidence is at least moderate and that we can, in general, endorse the recommendations made for laboratory and clinical practice.

Knowledge of Lp(a) could be particularly valuable in reclassification of patients at intermediate risk of ASCVD. Lp(a) should be measured in individuals with a personal or family history of premature ASCVD (or aortic valve stenosis), FH, or recurrent coronary events despite optimal LDL-c on diet and statins, with or without ezetimibe. Information on Lp(a) may guide more aggressive treatment of conventional risk factors or the need to assess subclinical atherosclerosis with cardiac CT scanning. Measurement of Lp(a) should employ an assay that is well standardized, isoform independent, and accordingly expressed based on the analysis and not the conversion factor as a molar and not mass concentration. We concur with the thresholds used to define Lp(a) risk in some guidelines but not others that set the level as >1.8 g/L. We do not agree that a single estimate of Lp(a) suffices to assess risk because plasma Lp(a) levels may vary with illness and certain medications, this being especially relevant when values are close to defined thresholds of risk that require action. More work is required to define Lp(a) risk thresholds in non-Caucasians, in whom interpretation and use of existing reference values should be guarded unless overridden by a clear clinical context.

The precise value of cascade testing first-degree relatives of an index case has not been demonstrated. However, it could help define and consolidate the family history of ASCVD and improve adherence to existing therapies in secondary preventation as well as to healthy lifestyle and behaviour in primary prevention. Elevated Lp(a) with a coexistent polygenic hypercholesterolaemia or familial combined hyperlipidaemia may mimic FH and should always be considered in patients who return a negative genetic test for FH [84]. A PCSK9 inhibitor could be used to lower Lp(a) in very high risk patients, but payers may need further convincing unless acquisition costs of these agents drop. Aspirin may be clinically indicated in patients with high Lp(a) who have extensive evidence of atherosclerosis on imaging, provided bleeding risk is minimal. We agree that there is no role for hormone-replacement therapy containing estrogen specifically for lowering Lp(a) in postmenopausal women and that apheresis may be indicated in all patients with high Lp(a) and progressive ASCVD. However, we do not agree that treatment with niacin has no role in managing high Lp(a), particularly in very high risk patients with polyvascular disease or young patients with FH and symptomatic ASCVD who have no access to PCSK9 inhibitors or apheresis.

**Figure 3:**
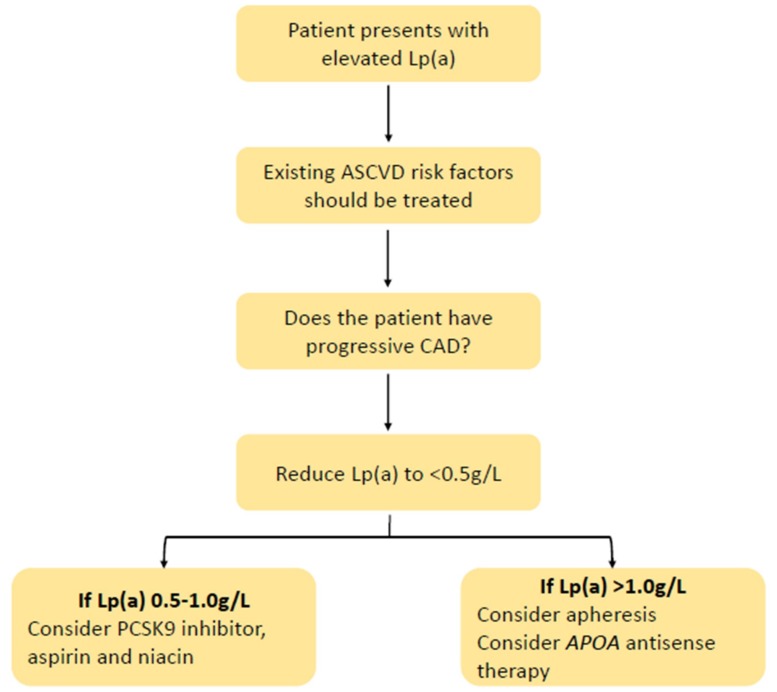
Proposed algorithm for the management of elevated lipoprotein(a). Lp(a) = lipoprotein (a), ASCVD = atherosclerotic cardiovascular disease, CAD = coronary artery disease, APOA = apolipoprotein (a) gene, PCSK9 = proprotein convertase subtilisin/kexin type 9.

The use of Lp(a) in the clinic will evidently require good judgement calls and balanced, shared decision making. The evidence levels and strength of recommendation for more extended measurmenet of Lp(a) will depend on clinical outcome trials employing specific therapies (such as apo(a) ASOs and siRNAs) targeted at high-risk patients (e.g., ASCVD, FH, or diabetes) with elevated Lp(a). A tentative algorithm based on expert opinion for the management of patients with elevated Lp(a) is suggested in Figure 3 [39]. As with FH, standardized models of care, registries, patient support groups, and codification for Lp(a) will eventually be required [22].

## Figures and Tables

**Table 1:** Summary of major lipid management guidelines on measurement and treatment of lipoprotein(a).

	Canadian Cardiovascular Society 2016 [104]	Mighty Medic Group 2017 [105]	AHA/ACC Group 2018 [103]	NLA 2019 [88]	EAS/ESC 2019 [106]	HEART-UK [107]
**Recommended measurement population**	Intermediate Framingham risk score or family history of premature CAD	Intermediate/high risk CVD patients with premature CVD, FH, or family history of premature CVD without elevated LDL-c or recurrent CVD with statin therapy	Family history of premature ASCVD or personal history of ASCVD not explained by major risk factors	Family or personal history of premature ASCVD, primary severe hypercholesterolaemia (LDL-c ≥ 190 mg/dL), or FH.	High CVD risk or strong family history of premature ASCVD, including premature CVD, FH, recurrent CVD despite optimal LLT, or ≥5% 10-year CVD risk. * Plasma levels should be measured at least once in a person’s life for risk stratification.	A personal or family history of premature ASCVD; 1 relative with Lp(a) > 200 nmol/L, FH, or genetic dyslipidaemia; CAVD; or increased 10-year risk of CVD
**Risk threshold**	>30 mg/dL	>30 mg/dL or >45 nmol/L	≥50 mg/dL or ≥125 nmol/L	≥50 mg/dL or ≥100 nmol/L * population dependent	State a level >180 mg/dL or >430 nmol/L equivalent to heterozygous FH	32–90 nmol/L, minor; 90–200 nmol/L, moderate; 200–400 nmol/L, high; >400 nmol/L, very high
**Recommended treatment**	Consideration of elevated Lp(a) in shared decision making	Niacin or, if refractory, selective apheresis.	None specified	None specified	None specified	Reduce overall ASCVD risk, control hyperlipidaemia, and consider apheresis

ACC, American College of Cardiology; AHA, American Heart Association; ASCVD, atherosclerotic cardiovascular disease; CAD, coronary artery disease; CAVD, calcific aortic valve disease; CVD, cardiovascular disease; EAS, European Atherosclerosis Society; ECS, European Society of Cariology; FH, familial hypercholesterolaemia; LDL-c, low-density lipoprotein cholesterol; Lp(a), lipoprotein (a); NLA, National Lipid Association; LLT, lipid lowering therapy; *, acknowledgement that Lp(a) varies between Caucasian and non-caucasian populations.

## References

[1] Berg K. (1963). A New Serum Type System in Man—The Lp System. Acta Pathol. Microbiol. Scand..

[2] McLean J.W., Tomlinson J.E., Kuang W.J., Eaton D.L., Chen E.Y., Fless G.M., Scanu A.M., Lawn R.M. (1987). cDNA sequence of human apolipoprotein(a) is homologous to plasminogen. Nature.

[3] Boffa M.B., Koschinsky M.L. (2019). Oxidized phospholipids as a unifying theory for lipoprotein(a) and cardiovascular disease. Nature reviews. Cardiology.

[4] Kostner K.M., Kostner G.M. (2017). Lipoprotein (a): A historical appraisal. J. Lipid Res..

[5] Weisel J.W., Nagaswami C., Woodhead J.L., Higazi A.A., Cain W.J., Marcovina S.M., Koschinsky M.L., Cines D.B., Bdeir K. (2001). The structure of lipoprotein(a) and ligand-induced conformational changes. Biochemistry.

[6] Tsimikas S.A. (2017). Test in Context: Lipoprotein(a): Diagnosis, Prognosis, Controversies, and Emerging Therapies. J. Am. Coll. Cardiol..

[7] Maranhao R.C., Carvalho P.O., Strunz C.C., Pileggi F. (2014). Lipoprotein (a): Structure, pathophysiology and clinical implications. Arq. Bras. Cardiol..

[8] Kostner K.M., Kostner G.M., Wierzbicki A.S. (2018). Is Lp(a) ready for prime time use in the clinic? A pros-and-cons debate. Atherosclerosis.

[9] Scipione C.A., Koschinsky M.L., Boffa M.B. (2018). Lipoprotein(a) in clinical practice: New perspectives from basic and translational science. Crit. Rev. Clin. Lab. Sci..

[10] Nicholls S.J., Nelson A.J. (2019). The time for lipoprotein(a) based intervention has arrived: Where will the light shine?. J. Thorac. Dis..

[11] Kronenberg F. (2019). Prediction of cardiovascular risk by Lp(a) concentrations or genetic variants within the LPA gene region. Clin. Res. Cardiol. Suppl..

[12] Lamon-Fava S., Jimenez D., Christian J.C., Fabsitz R.R., Reed T., Carmelli D., Castelli W.P., Ordovas J.M., Wilson P.W., Schaefer E.J. (1991). The NHLBI Twin Study: Heritability of apolipoprotein A-I, B, and low density lipoprotein subclasses and concordance for lipoprotein(a). Atherosclerosis.

[13] Austin M.A., Sandholzer C., Selby J.V., Newman B., Krauss R.M., Utermann G. (1992). Lipoprotein(a) in women twins: Heritability and relationship to apolipoprotein(a) phenotypes. Am. J. Hum. Genet..

[14] Kronenberg F. (2016). Human Genetics and the Causal Role of Lipoprotein(a) for Various Diseases. Cardiovasc. Drugs Ther..

[15] Hung M.Y., Tsimikas S. (2014). What is the ultimate test that lowering lipoprotein(a) is beneficial for cardiovascular disease and aortic stenosis?. Curr. Opin. Lipidol..

[16] Schmidt K., Noureen A., Kronenberg F., Utermann G. (2016). Structure, function, and genetics of lipoprotein (a). J. Lipid Res..

[17] Coassin S., Schönherr S., Weissensteiner H., Erhart G., Forer L., Losso J.L., Lamina C., Haun M., Utermann G., Paulweber B. (2019). A comprehensive map of single-base polymorphisms in the hypervariable LPA kringle IV type 2 copy number variation region. J. Lipid Res..

[18] Nordestgaard B.G., Langsted A. (2016). Lipoprotein (a) as a cause of cardiovascular disease: Insights from epidemiology, genetics, and biology. J. Lipid Res..

[19] Moriarty P.M., Varvel S.A., Gordts P.L., McConnell J.P., Tsimikas S. (2017). Lipoprotein(a) Mass Levels Increase Significantly According to APOE Genotype: An Analysis of 431 239 Patients. Arterioscler. Thromb. Vasc. Biol..

[20] Kritharides L., Nordestgaard B.G., Tybjærg-Hansen A., Kamstrup P.R., Afzal S. (2017). Effect of APOE epsilon Genotype on Lipoprotein(a) and the Associated Risk of Myocardial Infarction and Aortic Valve Stenosis. J. Clin. Endocrinol. Metab..

[21] Laschkolnig A., Kollerits B., Lamina C., Meisinger C., Rantner B., Stadler M., Peters A., Koenig W., Stöckl A., Dähnhardt D. (2014). Lipoprotein (a) concentrations, apolipoprotein (a) phenotypes, and peripheral arterial disease in three independent cohorts. Cardiovascular Research.

[22] Ellis K.L., Boffa M.B., Sahebkar A., Koschinsky M.L., Watts G.F. (2017). The renaissance of lipoprotein(a): Brave new world for preventive cardiology?. Prog. Lipid Res..

[23] Kostner K.M., Marz W., Kostner G.M. (2013). When should we measure lipoprotein (a)?. Eur. Heart J..

[24] Kronenberg F., Kuen E., Ritz E., Junker R., König P., Kraatz G., Lhotta K., Mann J.F., Müller G.A., Neyer U. (2000). Lipoprotein(a) serum concentrations and apolipoprotein(a) phenotypes in mild and moderate renal failure. J. Am. Soc. Nephrol..

[25] Tsimikas S. (2019). Potential Causality and Emerging Medical Therapies for Lipoprotein(a) and Its Associated Oxidized Phospholipids in Calcific Aortic Valve Stenosis. Circ. Res..

[26] Nestel P. (2019). Lipoprotein(a) Removal Still a Mystery. J. Am. Heart Assoc..

[27] McCormick S.P.A., Schneider W.J. (2019). Lipoprotein(a) catabolism: A case of multiple receptors. Pathology.

[28] Cantin B., Gagnon F., Moorjani S., Després J.P., Lamarche B., Lupien P.J., Dagenais G.R. (1998). Is lipoprotein(a) an independent risk factor for ischemic heart disease in men? The Quebec Cardiovascular Study. J. Am. Coll. Cardiol..

[29] Suk Danik J., Rifai N., Buring J.E., Ridker P.M. (2006). Lipoprotein(a), measured with an assay independent of apolipoprotein(a) isoform size, and risk of future cardiovascular events among initially healthy women. JAMA J. Am. Med. Assoc..

[30] Erqou S., Kaptoge S., Perry P.L., Di Angelantonio E., Thompson A., White I.R., Marcovina S.M., Collins R., Thompson S.G., Emerging Risk Factors Collaboration (2009). Lipoprotein(a) concentration and the risk of coronary heart disease, stroke, and nonvascular mortality. JAMA J. Am. Med. Assoc..

[31] von Eckardstein A., Schulte H., Cullen P., Assmann G. (2001). Lipoprotein(a) further increases the risk of coronary events in men with high global cardiovascular risk. J. Am. Coll. Cardiol..

[32] Sharrett A.R., Ballantyne C.M., Coady S.A., Heiss G., Sorlie P.D., Catellier D., Patsch W. (2001). Coronary heart disease prediction from lipoprotein cholesterol levels, triglycerides, lipoprotein(a), apolipoproteins A-I and B, and HDL density subfractions: The Atherosclerosis Risk in Communities (ARIC) Study. Circulation.

[33] Luc G., Bard J.M., Arveiler D., Ferrieres J., Evans A., Amouyel P., Fruchart J.C., Ducimetiere P. (2002). Lipoprotein (a) as a predictor of coronary heart disease: The PRIME Study. Atherosclerosis.

[34] Ariyo A.A., Thach C., Tracy R. (2003). Cardiovascular Health Study I. Lp(a) lipoprotein, vascular disease, and mortality in the elderly. N. Engl. J. Med..

[35] Rifai N., Ma J., Sacks F.M., Ridker P.M., Hernandez W.J., Stampfer M.J., Marcovina S.M. (2004). Apolipoprotein(a) size and lipoprotein(a) concentration and future risk of angina pectoris with evidence of severe coronary atherosclerosis in men: The Physicians’ Health Study. Clin. Chem..

[36] Kamstrup P.R., Benn M., Tybjaerg-Hansen A., Nordestgaard B.G. (2008). Extreme lipoprotein(a) levels and risk of myocardial infarction in the general population: The Copenhagen City Heart Study. Circulation.

[37] Seed M., Hoppichler F., Reaveley D., McCarthy S., Thompson G.R., Boerwinkle E., Utermann G. (1990). Relation of serum lipoprotein(a) concentration and apolipoprotein(a) phenotype to coronary heart disease in patients with familial hypercholesterolemia. N. Engl. J. Med..

[38] Danesh J., Collins R., Peto R. (2000). Lipoprotein(a) and coronary heart disease. Meta-analysis of prospective studies. Circulation.

[39] Nestel P.J., Barnes E.H., Tonkin A.M., Simes J., Fournier M., White H.D., Colquhoun D.M., Blankenberg S., Sullivan D.R. (2013). Plasma lipoprotein(a) concentration predicts future coronary and cardiovascular events in patients with stable coronary heart disease. Arterioscler. Thromb. Vasc. Biol..

[40] Willeit P., Ridker P.M., Nestel P.J., Simes J., Tonkin A.M., Pedersen T.R., Schwartz G.G., Olsson A.G., Colhoun H.M., Kronenberg F. (2018). Baseline and on-statin treatment lipoprotein(a) levels for prediction of cardiovascular events: Individual patient-data meta-analysis of statin outcome trials. Lancet.

[41] Verbeek R., Hoogeveen R.M., Langsted A., Stiekema L.C.A., Verweij S.L., Hovingh G.K., Wareham N.J., Khaw K.T., Boekholdt S.M., Nordestgaard B.G. (2018). Cardiovascular disease risk associated with elevated lipoprotein(a) attenuates at low low-density lipoprotein cholesterol levels in a primary prevention setting. Eur. Heart J..

[42] Rallidis L.S., Pavlakis G., Foscolou A., Kotakos C., Katsimardos A., Drosatos A., Zolindaki M., Panagiotakos D.B. (2018). High levels of lipoprotein (a) and premature acute coronary syndrome. Atherosclerosis.

[43] Foscolou A.G.E., Magriplis E., Naumovski N., Rallidis L., Matalas A.-L., Chrysohoou C., Tousoulis D., Pitsavos C., Panagiotakos D. (2019). The mediating role of Mediterranean diet on the association between Lp(a) levels and cardiovascular disease risk: A 10-year follow-up of the ATTICA study. Clin. Biochem..

[44] Boffa M.B., Stranges S., Klar N., Moriarty P.M., Watts G.F., Koschinsky M.L. (2018). Lipoprotein(a) and secondary prevention of atherothrombotic events: A critical appraisal. J. Clin. Lipidol..

[45] Muramatsu Y., Minami Y., Kato A., Katsura A., Sato T., Kakizaki R., Nemoto T., Hashimoto T., Fujiyoshi K., Meguro K. (2019). Lipoprotein (a) level is associated with plaque vulnerability in patients with coronary artery disease: An optical coherence tomography study. Int. J. Cardiol. Heart Vasc..

[46] Kronenberg F. (2019). Therapeutic lowering of lipoprotein(a): How much is enough?. Atherosclerosis.

[47] Madsen C.M., Kamstrup P.R., Langsted A., Varbo A., Nordestgaard B.G. (2019). Lp(a) (Lipoprotein[a])-Lowering by 50 mg/dL (105 nmol/L) May Be Needed to Reduce Cardiovascular Disease 20% in Secondary Prevention: A Population-Based Study. Arterioscler. Thromb. Vasc. Biol..

[48] Langsted A., Kamstrup P.R., Nordestgaard B.G. (2019). High lipoprotein(a) and high risk of mortality. Eur. Heart J..

[49] Kamstrup P.R., Tybjaerg-Hansen A., Nordestgaard B.G. (2014). Elevated lipoprotein(a) and risk of aortic valve stenosis in the general population. J. Am. Coll. Cardiol..

[50] Arsenault B.J., Boekholdt S.M., Dubé M.P., Rhéaume E., Wareham N.J., Khaw K.T., Sandhu M.S., Tardif J.C. (2014). Lipoprotein(a) levels, genotype, and incident aortic valve stenosis: A prospective Mendelian randomization study and replication in a case-control cohort. Circ. Cardiovasc. Genet..

[51] Vongpromek R., Bos S., Ten Kate G.J., Yahya R., Verhoeven A.J., de Feyter P.J., Kronenberg F., Roeters van Lennep J.E., Sijbrands E.J., Mulder M.T. (2015). Lipoprotein(a) levels are associated with aortic valve calcification in asymptomatic patients with familial hypercholesterolaemia. J. Intern. Med..

[52] Capoulade R., Chan K.L., Yeang C., Mathieu P., Bossé Y., Dumesnil J.G., Tam J.W., Teo K.K., Mahmut A., Yang X. (2015). Oxidized Phospholipids, Lipoprotein(a), and Progression of Calcific Aortic Valve Stenosis. J. Am. Coll. Cardiol..

[53] Capoulade R., Yeang C., Chan K.L., Pibarot P., Tsimikas S. (2018). Association of Mild to Moderate Aortic Valve Stenosis Progression With Higher Lipoprotein(a) and Oxidized Phospholipid Levels: Secondary Analysis of a Randomized Clinical Trial. JAMA Cardiol..

[54] Zheng K.H., Tsimikas S., Pawade T., Kroon J., Jenkins W.S.A., Doris M.K., White A.C., Timmers N.K.L.M., Hjortnaes J., Rogers M.A. (2019). Lipoprotein(a) and Oxidized Phospholipids Promote Valve Calcification in Patients With Aortic Stenosis. J. Am. Coll. Cardiol..

[55] Golledge J., Ward N.C., Watts G.F. (2019). Lipid management in people with peripheral artery disease. Curr. Opin. Lipidol..

[56] Volpato S., Vigna G.B., McDermott M.M., Cavalieri M., Maraldi C., Lauretani F., Bandinelli S., Zuliani G., Guralnik J.M., Fellin R. (2010). Lipoprotein(a), inflammation, and peripheral arterial disease in a community-based sample of older men and women (the InCHIANTI study). Am. J. Cardiol..

[57] Gurdasani D., Sjouke B., Tsimikas S., Hovingh G.K., Luben R.N., Wainwright N.W., Pomilla C., Wareham N.J., Khaw K.T., Boekholdt S.M. (2012). Lipoprotein(a) and risk of coronary, cerebrovascular, and peripheral artery disease: The EPIC-Norfolk prospective population study. Arterioscler. Thromb. Vasc. Biol..

[58] Anand S.S., Yusuf S., Vuksan V., Devanesen S., Teo K.K., Montague P.A., Kelemen L., Yi C., Lonn E., Gerstein H. (2000). Differences in risk factors, atherosclerosis, and cardiovascular disease between ethnic groups in Canada: The Study of Health Assessment and Risk in Ethnic groups (SHARE). Lancet.

[59] Virani S.S., Brautbar A., Davis B.C., Nambi V., Hoogeveen R.C., Sharrett A.R., Coresh J., Mosley T.H., Morrisett J.D., Catellier D.J. (2012). Associations between lipoprotein(a) levels and cardiovascular outcomes in black and white subjects: The Atherosclerosis Risk in Communities (ARIC) Study. Circulation.

[60] Waldeyer C., Makarova N., Zeller T., Schnabel R.B., Brunner F.J., Jørgensen T., Linneberg A., Niiranen T., Salomaa V., Jousilahti P. (2017). Lipoprotein(a) and the risk of cardiovascular disease in the European population: Results from the BiomarCaRE consortium. Eur. Heart J..

[61] Paré G., Çaku A., McQueen M., Anand S.S., Enas E., Clarke R., Boffa M.B., Koschinsky M., Wang X., Yusuf S. (2019). Lipoprotein(a) Levels and the Risk of Myocardial Infarction Among 7 Ethnic Groups. Circulation.

[62] Cai G., Huang Z., Zhang B., Yu L., Li L. (2019). Elevated lipoprotein (a) levels are associated with the acute myocardial infarction in patients with normal low-density lipoprotein cholesterol levels. Biosci. Rep..

[63] Steffen B.T., Duprez D., Bertoni A.G., Guan W., Tsai M.Y. (2018). Lp(a) [Lipoprotein(a)]-Related Risk of Heart Failure Is Evident in Whites but Not in Other Racial/Ethnic Groups. Arterioscler. Thromb. Vasc. Biol..

[64] Khera A.V., Everett B.M., Caulfield M.P., Hantash F.M., Wohlgemuth J., Ridker P.M., Mora S. (2014). Lipoprotein(a) concentrations, rosuvastatin therapy, and residual vascular risk: An analysis from the JUPITER Trial (Justification for the Use of Statins in Prevention: An Intervention Trial Evaluating Rosuvastatin). Circulation.

[65] Tsimikas S., Gordts P.L.S.M., Nora C., Yeang C., Witztum J.L. (2019). Statin therapy increases lipoprotein(a) levels. Eur. Heart J..

[66] Yahya R., Berk K., Verhoeven A., Bos S., van der Zee L., Touw J., Erhart G., Kronenberg F., Timman R., Sijbrands E. (2019). Statin treatment increases lipoprotein(a) levels in subjects with low molecular weight apolipoprotein(a) phenotype. Atherosclerosis.

[67] Paige E., Masconi K.L., Tsimikas S., Kronenberg F., Santer P., Weger S., Willeit J., Kiechl S., Willeit P. (2017). Lipoprotein(a) and incident type-2 diabetes: Results from the prospective Bruneck study and a meta-analysis of published literature. Cardiovasc. Diabetol..

[68] Zhang H.W., Zhao X., Guo Y.L., Gao Y., Zhu C.G., Wu N.Q., Li J.J. (2018). Elevated lipoprotein (a) levels are associated with the presence and severity of coronary artery disease in patients with type 2 diabetes mellitus. Nutrition, metabolism, and cardiovascular diseases. Nutr. Metab. Cardiovasc. Dis..

[69] Jin J.L., Cao Y.X., Zhang H.W., Sun D., Hua Q., Li Y.F., Guo Y.L., Wu N.Q., Zhu C.G., Gao Y. (2019). Lipoprotein(a) and Cardiovascular Outcomes in Patients With Coronary Artery Disease and Prediabetes or Diabetes. Diabetes Care.

[70] Saeed A., Sun W., Agarwala A., Virani S.S., Nambi V., Coresh J., Selvin E., Boerwinkle E., Jones P.H., Ballantyne C.M. (2019). Lipoprotein(a) levels and risk of cardiovascular disease events in individuals with diabetes mellitus or prediabetes: The Atherosclerosis Risk in Communities study. Atherosclerosis.

[71] Werba J.P., Safa O., Gianfranceschi G., Michelagnoli S., Sirtori C.R., Franceschini G. (1993). Plasma triglycerides and lipoprotein(a): Inverse relationship in a hyperlipidemic Italian population. Atherosclerosis.

[72] Consortium C.A.D., Deloukas P., Kanoni S., Willenborg C., Farrall M., Assimes T.L., Thompson J.R., Ingelsson E., Saleheen D., Erdmann J. (2013). Large-scale association analysis identifies new risk loci for coronary artery disease. Nat. Genet..

[73] Clarke R., Peden J.F., Hopewell J.C., Kyriakou T., Goel A., Heath S.C., Parish S., Barlera S., Franzosi M.G., Rust S. (2009). Genetic variants associated with Lp(a) lipoprotein level and coronary disease. N. Engl. J. Med..

[74] Thanassoulis G., Campbell C.Y., Owens D.S., Smith J.G., Smith A.V., Peloso G.M., Kerr K.F., Pechlivanis S., Budoff M.J., Harris T.B. (2013). Genetic associations with valvular calcification and aortic stenosis. N. Engl. J. Med..

[75] Kamstrup P.R., Tybjaerg-Hansen A., Steffensen R., Nordestgaard B.G. (2009). Genetically elevated lipoprotein(a) and increased risk of myocardial infarction. JAMA J. Am. Med. Assoc..

[76] Coassin S., Erhart G., Weissensteiner H., Eca Guimarães de Araújo M., Lamina C., Schönherr S., Forer L., Haun M., Losso J.L., Köttgen A. (2017). A novel but frequent variant in LPA KIV-2 is associated with a pronounced Lp(a) and cardiovascular risk reduction. Eur. Heart J..

[77] Noureen A., Fresser F., Utermann G., Schmidt K. (2015). Sequence variation within the KIV-2 copy number polymorphism of the human LPA gene in African, Asian, and European populations. PLoS ONE.

[78] Saleheen D., Haycock P.C., Zhao W., Rasheed A., Taleb A., Imran A., Abbas S., Majeed F., Akhtar S., Qamar N. (2017). Apolipoprotein(a) isoform size, lipoprotein(a) concentration, and coronary artery disease: A mendelian randomisation analysis. Lancet Diabetes Endocrinol..

[79] Kamstrup P.R., Nordestgaard B.G. (2016). Elevated Lipoprotein(a) Levels, LPA Risk Genotypes, and Increased Risk of Heart Failure in the General Population. JACC Heart Fail..

[80] Lamina C., Kronenberg F. (2019). Lp GC. Estimation of the Required Lipoprotein(a)-Lowering Therapeutic Effect Size for Reduction in Coronary Heart Disease Outcomes: A Mendelian Randomization Analysis. JAMA Cardiol..

[81] Nordestgaard B.G., Chapman M.J., Ray K., Borén J., Andreotti F., Watts G.F., Ginsberg H., Amarenco P., Catapano A., Descamps O.S. (2010). Lipoprotein(a) as a cardiovascular risk factor: Current status. Eur. Heart J..

[82] Burgess S., Ference B.A., Staley J.R., Freitag D.F., Mason A.M., Nielsen S.F., Willeit P., Young R., Surendran P., Karthikeyan S. (2018). Association of LPA Variants With Risk of Coronary Disease and the Implications for Lipoprotein(a)-Lowering Therapies: A Mendelian Randomization Analysis. JAMA Cardiol..

[83] Najam O., Ray K.K. (2019). Lp(a) and cardiovascular disease-Has the phoenix finally risen from the ashes?. Eur. Heart J..

[84] Ellis K.L., Hooper A.J., Burnett J.R., Watts G.F. (2016). Progress in the care of common inherited atherogenic disorders of apolipoprotein B metabolism. Nat. Rev. Endocrinol..

[85] (2019). The challenges of measuring Lp(a): A fight against Hydra?. Atherosclerosis.

[86] Dati F., Tate J.R., Marcovina S.M., Steinmetz A., International Federation of Clinical Chemistry and Laboratory Medicine, IFCC Working Group for Lipoprotein(a) Assay Standardization (2004). First WHO/IFCC International Reference Reagent for Lipoprotein(a) for Immunoassay—Lp(a) SRM 2B. Clin. Chem. Lab. Med..

[87] Marcovina S.M., Albers J.J., Scanu A.M., Kennedy H., Giaculli F., Berg K., Couderc R., Dati F., Rifai N., Sakurabayashi I. (2000). Use of a reference material proposed by the International Federation of Clinical Chemistry and Laboratory Medicine to evaluate analytical methods for the determination of plasma lipoprotein(a). Clin. Chem..

[88] Wilson D.P., Jacobson T.A., Jones P.H., Koschinsky M.L., McNeal C.J., Nordestgaard B.G., Orringer C.E. (2019). Use of Lipoprotein(a) in clinical practice: A biomarker whose time has come. A scientific statement from the National Lipid Association. J. Clin. Lipidol..

[89] Scharnagl H., Stojakovic T., Dieplinger B., Dieplinger H., Erhart G., Kostner G.M., Herrmann M., März W., Grammer T.B. (2019). Comparison of lipoprotein (a) serum concentrations measured by six commercially available immunoassays. Atherosclerosis.

[90] Marcovina S.M., Zhang Z.H., Gaur V.P., Albers J.J. (1993). Identification of 34 apolipoprotein(a) isoforms: Differential expression of apolipoprotein(a) alleles between American blacks and whites. Biochem. Biophys. Res. Commun..

[91] Lassman M.E., McLaughlin T.M., Zhou H., Pan Y., Marcovina S.M., Laterza O., Roddy T.P. (2014). Simultaneous quantitation and size characterization of apolipoprotein(a) by ultra-performance liquid chromatography/mass spectrometry. Rapid Commun. Mass Spectrom..

[92] Ellis K.L., Watts G.F. (2018). Is Lipoprotein(a) Ready for Prime-Time Use in the Clinic?. Cardiol. Clin..

[93] Marcovina S.M., Albers J.J. (2016). Lipoprotein (a) measurements for clinical application. J. Lipid Res..

[94] Tsimikas S., Fazio S., Ferdinand K.C., Ginsberg H.N., Koschinsky M.L., Marcovina S.M., Moriarty P.M., Rader D.J., Remaley A.T., Reyes-Soffer G. (2018). NHLBI Working Group Recommendations to Reduce Lipoprotein(a)-Mediated Risk of Cardiovascular Disease and Aortic Stenosis. J. Am. Coll. Cardiol..

[95] Ellis K.L., Chakraborty A., Moses E.K., Watts G.F. (2019). To test, or not to test: That is the question for the future of lipoprotein(a). Expert Rev. Cardiovasc. Ther..

[96] Takami S., Yamashita S., Kihara S., Ishigami M., Takemura K., Kume N., Kita T., Matsuzawa Y. (1998). Lipoprotein(a) enhances the expression of intercellular adhesion molecule-1 in cultured human umbilical vein endothelial cells. Circulation.

[97] Cho T., Jung Y., Koschinsky M.L. (2008). Apolipoprotein(a), through its strong lysine-binding site in KIV(10’), mediates increased endothelial cell contraction and permeability via a Rho/Rho kinase/MYPT1-dependent pathway. J. Biol. Chem..

[98] Bouchareb R., Mahmut A., Nsaibia M.J., Boulanger M.C., Dahou A., Lépine J.L., Laflamme M.H., Hadji F., Couture C., Trahan S. (2015). Autotaxin Derived From Lipoprotein(a) and Valve Interstitial Cells Promotes Inflammation and Mineralization of the Aortic Valve. Circulation.

[99] Riches K., Porter K.E. (2012). Lipoprotein(a): Cellular Effects and Molecular Mechanisms. Cholesterol.

[100] Tsironis L.D., Mitsios J.V., Milionis H.J., Elisaf M., Tselepis A.D. (2004). Effect of lipoprotein (a) on platelet activation induced by platelet-activating factor: Role of apolipoprotein (a) and endogenous PAF-acetylhydrolase. Cardiovasc. Res..

[101] Boffa M.B., Koschinsky M.L. (2016). Lipoprotein (a): Truly a direct prothrombotic factor in cardiovascular disease?. J. Lipid Res..

[102] Catapano A.L., Graham I., De Backer G., Wiklund O., Chapman M.J., Drexel H., Hoes A.W., Jennings C.S., Landmesser U., Pedersen T.R. (2016). 2016 ESC/EAS Guidelines for the Management of Dyslipidaemias. Eur. Heart J..

[103] Grundy S.M., Stone N.J., Bailey A.L., Beam C., Birtcher K.K., Blumenthal R.S., Braun L.T., de Ferranti S., Faiella-Tommasino J., Forman D.E., Goldberg R. (2018). AHA/ACC/AACVPR/AAPA/ABC/ACPM/ADA/AGS/APhA/ASPC/NLA/PCNA guideline on the management of blood cholesterol: A report of the American College of Cardiology/American Heart Association Task Force on Clinical Practice Guidelines. Circulation.

[104] Anderson T.J., Gregoire J., Pearson G.J., Barry A.R., Couture P., Dawes M., Francis G.A., Genest J., Grover S., Gupta M. (2016). 2016 Canadian Cardiovascular Society Guidelines for the Management of Dyslipidemia for the Prevention of Cardiovascular Disease in the Adult. Can. J. Cardiol..

[105] Stefanutti C., Julius U., Watts G.F., Harada-Shiba M., Cossu M., Schettler V.J., De Silvestro G., Soran H., Van Lennep J.R., Pisciotta L. (2017). Toward an international consensus-Integrating lipoprotein apheresis and new lipid-lowering drugs. J. Clin. Lipidol..

[106] Mach F., Baigent C., Catapano A.L., Koskinas K.C., Casula M., Badimon L., Chapman M.J., de Backer G.G., Delgado V. (2019). (EAS) TTFftmodotESoCEaEAS. 2019 ESC/EAS Guidelines for the management of dyslipidaemias: Lipid modification to reduce cardiovascular risk. Eur. Heart J..

[107] Cegla J., Neely R.D.G., France M., Ferns G., Byrne C.D., Halcox J., Datta D., Capps N., Shoulders C., Qureshi N. (2019). HEART UK Medical, Scientific and Research Committee. HEART UK consensus statement on Lipoprotin(a): A call to action. Atherosclerosis.

[108] Afshar M., Pilote L., Dufresne L., Engert J.C., Thanassoulis G. (2016). Lipoprotein(a) Interactions With Low-Density Lipoprotein Cholesterol and Other Cardiovascular Risk Factors in Premature Acute Coronary Syndrome (ACS). J. Am. Heart Assoc..

[109] Chieng D., Pang J., Ellis K.L., Hillis G.S., Watts G.F., Schultz C.J. (2018). Elevated lipoprotein(a) and low-density lipoprotein cholesterol as predictors of the severity and complexity of angiographic lesions in patients with premature coronary artery disease. J. Clin. Lipidol..

[110] Ye Z., Haycock P.C., Gurdasani D., Pomilla C., Boekholdt S.M., Tsimikas S., Khaw K.T., Wareham N.J., Sandhu M.S., Forouhi N.G. (2014). The association between circulating lipoprotein(a) and type 2 diabetes: Is it causal?. Diabetes.

[111] Zawacki A.W., Dodge A., Woo K.M., Ralphe J.C., Peterson A.L. (2018). In pediatric familial hypercholesterolemia, lipoprotein(a) is more predictive than LDL-C for early onset of cardiovascular disease in family members. J. Clin. Lipidol..

[112] Ellis K.L., Perez de Isla L., Alonso R., Fuentes F., Watts G.F., Mata P. (2019). Value of Measuring Lipoprotein(a) During Cascade Testing for Familial Hypercholesterolemia. J. Am. Coll. Cardiol..

[113] Hopewell J.C., Haynes R., Baigent C. (2018). The role of lipoprotein (a) in chronic kidney disease. J. Lipid Res..

[114] Bajaj A., Damrauer S.M., Anderson A.H., Xie D., Budoff M.J., Go A.S., He J., Lash J.P., Ojo A., Post W.S. (2017). Lipoprotein(a) and Risk of Myocardial Infarction and Death in Chronic Kidney Disease: Findings From the CRIC Study (Chronic Renal Insufficiency Cohort). Arterioscler. Thromb. Vasc. Biol.

[115] Shah P., Bajaj S., Virk H., Bikkina M., Shamoon F. (2015). Rapid Progression of Coronary Atherosclerosis: A Review. Thrombosis.

[116] Cook N.R., Mora S., Ridker P.M. (2018). Lipoprotein(a) and Cardiovascular Risk Prediction Among Women. J. Am. Coll. Cardiol..

[117] O’Donoghue M.L., Giugliano R.P., Stroes E.S.G., Kanevsky E., Gouni-Berthold I., Im K.A., Pineda A.L., Wasserman S.M., Ceska R., Ezhov M.V. (2018). Lipoprotein(a), PCSK9 inhibition and cardiovascular risk: Insights from the FOURIER trial. Circulation.

[118] Chennamsetty I., Kostner K.M., Claudel T., Vinod M., Frank S., Weiss T.S., Trauner M., Kostner G.M. (2012). Nicotinic acid inhibits hepatic APOA gene expression: Studies in humans and in transgenic mice. J. Lipid Res..

[119] (1975). Clofibrate and niacin in coronary heart disease. JAMA J. Am. Med. Assoc..

[120] Tuck C.H., Holleran S., Berglund L. (1997). Hormonal regulation of lipoprotein(a) levels: Effects of estrogen replacement therapy on lipoprotein(a) and acute phase reactants in postmenopausal women. Arterioscler. Thromb. Vasc. Biol..

[121] Henriksson P., Angelin B., Berglund L. (1992). Hormonal regulation of serum Lp (a) levels. Opposite effects after estrogen treatment and orchidectomy in males with prostatic carcinoma. J. Clin. Investig..

[122] Sahebkar A., Serban M.C., Penson P., Gurban C., Ursoniu S., Toth P.P., Jones S.R., Lippi G., Kotani K., Kostner K. (2017). The Effects of Tamoxifen on Plasma Lipoprotein(a) Concentrations: Systematic Review and *Meta Anal*. Drugs.

[123] Momtazi-Borojeni A.A., Katsiki N., Pirro M., Banach M., Rasadi K.A., Sahebkar A. (2019). Dietary natural products as emerging lipoprotein(a)-lowering agents. J. Cell Physiol..

[124] Jaeger B.R., Richter Y., Nagel D., Heigl F., Vogt A., Roeseler E., Parhofer K., Ramlow W., Koch M., Utermann G. (2009). Longitudinal cohort study on the effectiveness of lipid apheresis treatment to reduce high lipoprotein(a) levels and prevent major adverse coronary events. Nat. Clin. Pract. Cardiovasc Med..

[125] Leebmann J., Roeseler E., Julius U., Heigl F., Spitthoever R., Heutling D., Breitenberger P., Maerz W., Lehmacher W., Heibges A. (2013). Lipoprotein apheresis in patients with maximally tolerated lipid-lowering therapy, lipoprotein(a)-hyperlipoproteinemia, and progressive cardiovascular disease: Prospective observational multicenter study. Circulation.

[126] Roeseler E., Julius U., Heigl F., Spitthoever R., Heutling D., Breitenberger P., Leebmann J., Lehmacher W., Kamstrup P.R., Nordestgaard B.G. (2016). Lipoprotein Apheresis for Lipoprotein(a)-Associated Cardiovascular Disease: Prospective 5 Years of Follow-Up and Apolipoprotein(a) Characterization. Arterioscler. Thromb. Vasc. Biol..

[127] Thompson G., Parhofer K.G. (2019). Current Role of Lipoprotein Apheresis. Curr. Atheroscler. Rep..

[128] van Capelleveen J.C., van der Valk F.M., Stroes E.S. (2016). Current therapies for lowering lipoprotein (a). J. Lipid Res..

[129] Stiekema L.C.A., Stroes E.S.G., Verweij S.L., Kassahun H., Chen L., Wasserman S.M., Sabatine M.S., Mani V., Fayad Z.A. (2018). Persistent arterial wall inflammation in patients with elevated lipoprotein(a) despite strong low-density lipoprotein cholesterol reduction by proprotein convertase subtilisin/kexin type 9 antibody treatment. Eur. Heart J..

[130] Ray K.K., Vallejo-Vaz A.J., Ginsberg H.N., Davidson M.H., Louie M.J., Bujas-Bobanovic M., Minini P., Eckel R.H., Cannon C.P. (2019). Lipoprotein(a) reductions from PCSK9 inhibition and major adverse cardiovascular events: Pooled analysis of alirocumab phase 3 trials. Atherosclerosis.

[131] Warden B.A., Minnier J., Watts G.F., Fazio S., Shapiro M.D. (2019). Impact of PCSK9 inhibitors on plasma lipoprotein(a) concentrations with or without a background of niacin therapy. J. Clin. Lipidol..

[132] Tsimikas S., Viney N.J., Hughes S.G., Singleton W., Graham M.J., Baker B.F., Burkey J.L., Yang Q., Marcovina S.M., Geary R.S. (2015). Antisense therapy targeting apolipoprotein(a): A randomised, double-blind, placebo-controlled phase 1 study. Lancet.

[133] Viney N.J., van Capelleveen J.C., Geary R.S., Xia S., Tami J.A., Yu R.Z., Marcovina S.M., Hughes S.G., Graham M.J., Crooke R.M. (2016). Antisense oligonucleotides targeting apolipoprotein(a) in people with raised lipoprotein(a): Two randomised, double-blind, placebo-controlled, dose-ranging trials. Lancet.

[134] Mencarelli A., Fiorucci S. (2010). FXR an emerging therapeutic target for the treatment of atherosclerosis. J Cell Mol Med.

[135] Chennamsetty I., Claudel T., Kostner K.M., Baghdasaryan A., Kratky D., Levak-Frank S., Frank S., Gonzalez F.J., Trauner M., Kostner G.M. (2011). Farnesoid X receptor represses hepatic human APOA gene expression. J. Clin. Investig..

[136] Thanassoulis G. (2019). Screening for High Lipoprotein(a). Circulation.

[137] Vassiliou V.S., Flynn P.D., Raphael C.E., Newsome S., Khan T., Ali A., Halliday B., Studer Bruengger A., Malley T., Sharma P. (2017). Lipoprotein(a) in patients with aortic stenosis: Insights from cardiovascular magnetic resonance. PLoS ONE.

